# Drug rash with eosinophilia and systemic symptoms and severe renal injury induced by proton pump inhibitor therapy: A case report

**DOI:** 10.1097/MD.0000000000022509

**Published:** 2020-10-16

**Authors:** Qien He, Guanghui Ying, Xiapei Fei, Chenqin Zha, Zhaogui Chen, Yishu Bao, Jiaorong Long, Zhujun Wang, Xuelin He, Min Xia

**Affiliations:** a Department of Nephrology, Beilun People's Hospital, Ning Bo; b Kidney Disease Center, The First Affiliated Hospital, College of Medicine, Zhejiang University; c Key Laboratory of Kidney Disease Prevention and Control Technology, Zhejiang Province; d The Third Grade Laboratory under the National State, Administration of Traditional Chinese Medicine, Hangzhou, Zhejiang, China.

**Keywords:** DRESS, severe renal injury, PPI

## Abstract

**Introduction:**

Proton pump inhibitors (PPIs) are widely prescribed and generally well tolerated but can rarely cause severe allergic reactions, such as drug rash with eosinophilia and systemic symptoms (DRESS). We report a case of DRESS and renal injury induced by PPIs, and describe the therapeutic process.

**Patient concerns:**

The patient was a 66-year-old female who complained of fever, pruritus, desquamation, erythema multiforme, and anuria caused by omeprazole taken for 2 weeks to treat abdominal distention.

**Diagnosis:**

The clinical history revealed a similar episode of PPI-induced fever, eosinophilia, and acute kidney injury more than 1 year ago. The present laboratory tests revealed eosinophilia and oliguric renal failure. The renal biopsy was performed subsequently and proved the diagnosis of PPI-induced DRESS.

**Interventions:**

After the suspected diagnosis of PPI-induced DRESS, omeprazole was discontinued and methylprednisolone infusion (40 mg qd) was initiated. Because of oliguric renal failure, the patient received intermittent hemodialysis.

**Outcomes:**

The patient initially responded to omeprazole discontinuation, hemodialysis, and glucocorticoids but later died from severe infection during the tapering of glucocorticoid therapy.

**Conclusion:**

Clinicians should remain on high alert for potential life-threatening complications when prescribing PPIs. If unexplained renal injury develops in a patient taking a PPI, renal biopsy may help in identifying the pathogenesis and might facilitate timely intervention.

## 1 Introduction

Drug rash with eosinophilia and systemic symptoms (DRESS) is a severe delayed-type hypersensitivity syndrome associated with erythema multiforme-type drug eruption and life-threatening systemic manifestations that primarily involve the liver, kidneys, lungs, and pancreas.^[1]^ DRESS is clinically divided into immediate and delayed types. Immediate-type DRESS is considered an immunoglobulin E (IgE)-mediated type-I hypersensitivity reaction,^[2–4]^ whereas delayed-type DRESS is thought to involve a T cell-mediated hypersensitivity reaction.^[5]^ Although proton pump inhibitors (PPIs) rarely cause DRESS,^[6]^ these drugs have been reported to cause hypersensitivity reactions in 0.2% to 3% of cases,^[7]^ with a mortality rate of 10%.^[8]^ Previous studies have reported DRESS syndrome induced by a variety of PPIs, including rabeprazole, pantoprazole, esomeprazole, lansoprazole, and omeprazole.^[5,9–12]^

Here, we present the case of a woman who died following the repeat occurrence of DRESS syndrome and renal failure caused by a 2-week course of omeprazole taken to treat symptoms of abdominal distention. The patient had developed DRESS syndrome in response to PPI therapy the previous year but had not received a renal biopsy or regular follow-up after the first episode. This case highlights the need for clinicians to remain on high alert for potential life-threatening complications when prescribing PPIs and to consider renal biopsy in cases of unexplained renal injury during therapy with a PPI.

## 2 Case presentation

**Figure 1:**
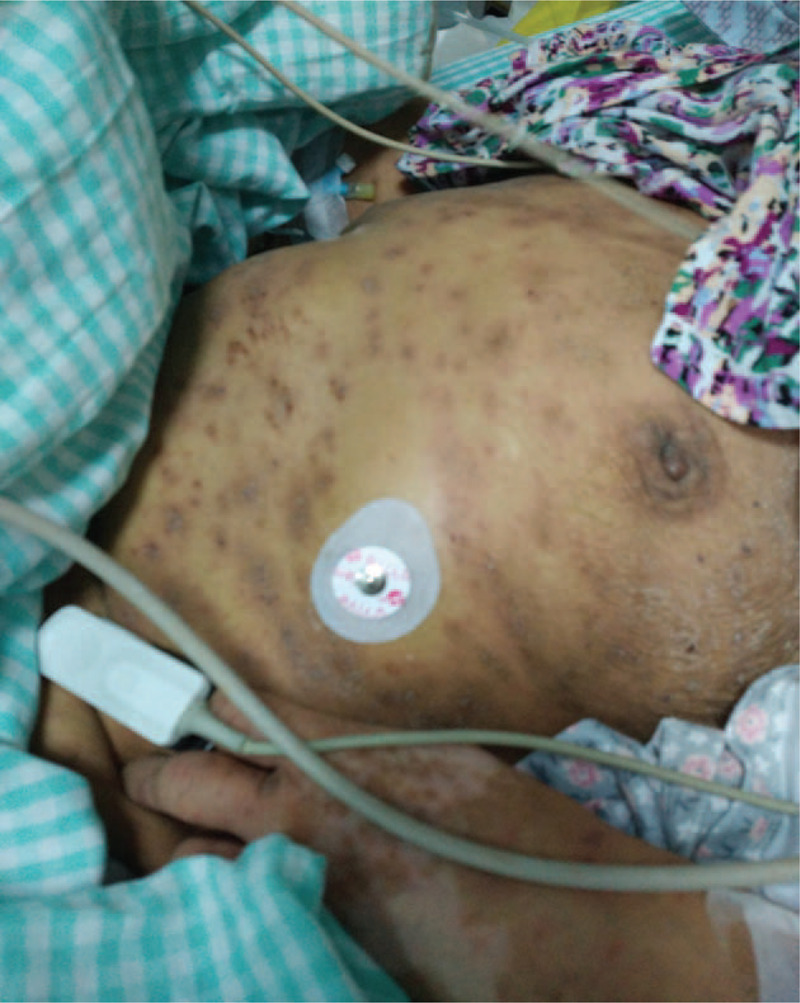
Pruritus and rash over the whole body (including the limbs) of the patient, with features that include desquamation, papules, macules, partially integrated blisters, and scabs.

On August 28, 2015, a 66-year-old Chinese woman was admitted to Beilun People's Hospital (Ningbo, China) with symptoms of fever, rash, chest tightness, and anuria, and a provisional diagnosis of renal failure was made. The patient had started a course of oral omeprazole (20 mg qd; purchased from a drug store) 2 weeks before admission due to symptoms of abdominal distension. In the week prior to admission, the patient had gradually developed pruritus and a rash over her whole body (including the limbs), with features that included desquamation, papules, macules, partially integrated blisters, and scabs (Fig. 1).

The patient denied a history of chronic diseases, such as hypertension or diabetes, or the long-term use of traditional Chinese medicines (a potential cause of renal injury). However, the patient had developed DRESS and acute renal failure after PPI therapy more than 1 year before the current admission. Specific details of the previous episode of DRESS are as follows: On July 1, 2014, the patient was admitted to a local hospital because of cough and gastrointestinal symptoms, such as nausea and vomiting. No abnormalities were detected in basic renal function tests (serum creatinine concentration, 75 μmol/L) or in a routine urine test. Three days later, the patient developed fever, cough, abdominal pain, nausea, and vomiting. She was treated initially with omeprazole infusion (40 mg qd) and then with long-term (>1 month) PPI therapy that included omeprazole, pantoprazole, and esomeprazole, in succession. Subsequently, the patient developed a fever (>38.5 °C) without any rash. Routine blood tests revealed an increase in eosinophil count (as high as 2.55 × 10^9^/L; normal range, 0.1–0.4 × 10^9^/L) and eosinophil proportion (as high as 20.1%; normal range, 0.4%–3%). A routine urine test was positive for leukocytes (an eosinophil granulocyte test was not conducted) but negative for proteinuria and microscopic hematuria. The patient received continuous renal replacement therapy (CRRT) for oliguria and acute renal failure for more than 2 weeks, which restored her serum creatinine level to 100 μmol/L. However, the patient did not undergo a renal biopsy after the completion of CRRT and was not followed-up during the subsequent year.

The blood hematology and biochemistry findings on admission of the patient to our hospital were as follows: leukocyte count, 8.7 × 10^9^/L (normal range, 3.6–11.0 × 10^9^/L), eosinophil count, 0.83 × 10^9^/L (normal range, 0.1–0.4 × 10^9^/L), eosinophil proportion, 9.6% (normal range, 0.4%–3%), hemoglobin concentration, 56 g/L (normal range, 115–165 g/L), platelet count, 108 × 10^9^/L (normal range, 140–400 × 10^9^/L), serum creatinine concentration, 1181 μmol/L (normal range, 45–84 μmol/L), blood urea nitrogen, 57.9 mmol/L (normal range, 2.5–7.0 mmol/L), and K^+^ concentration 7.17 mmol/L (normal range, 3.5–5.5 mmol/L). Erythrocyte sedimentation rate was normal. Arterial blood gas analysis showed severe metabolic acidosis (pH, 7.03 [normal range, 7.36–7.44]; HCO_3_^−^, 2 mmol/L [normal range, 22–28 mmol/L]; base excess, 28 mmol/L [normal range, ± 2 mmol/L]); and hyponatremia (Na^+^, 123 mmol/L; normal range, 133–146 mmol/L). Color Doppler ultrasonography of the urinary system showed that the sizes of the two kidneys were 102 × 48 mm and 98 × 47 mm, respectively, and that the bilateral renal cortex was about 4 mm in thickness. Routine urine tests were positive for glucose (2+), protein (+), and red blood cells (+ or 2+). The concentration of urinary α1-microglobulin was 21.9 mg/dL, and that of urinary micro-albumin was 36 mg/dL. Tests for the presence of anti-myeloperoxidase (MPO) antibody in peripheral blood produced weak positive results on two occasions. Tests for perinuclear and cytoplasmic anti-neutrophil cytoplasmic antibodies (p-ANCA and c-ANCA) were negative. Blood culture and echocardiography showed no abnormalities, and there was no swelling of the superficial lymph nodes. The patient subsequently received intermittent hemodialysis.

A diagnosis of PPI-induced DRESS was suspected based on the rapid development of fever, rash, and acute renal failure after the patient had started taking omeprazole. The skin lesions were also considered to be a drug rash upon consultation with dermatologists.

The criteria suggested by the Registry of Severe Cutaneous Adverse Reaction (RegiSCAR) are widely used for assessing the probability of an adverse drug reaction.^[13]^ According to this scoring system, a case for DRESS can be estimated as follows: fever ≥38.5 °C (0 point), enlarged lymph nodes (0 point), eosinophilia >1.5 × 10^9^/L (2 point), atypical lymphocytes (0 point), organ involvement (2 points), resolution ≥15 days (0 point), skin rash >50% of the body surface area (1 point), skin rash suggesting DRESS (1 point), organ involvement including the kidneys and digestive system (2 point), and negative evaluation of other potential causes (1 point). The RegiSCAR score of 9 points indicates that the case presented here was definitely of DRESS.

**Figure 2:**
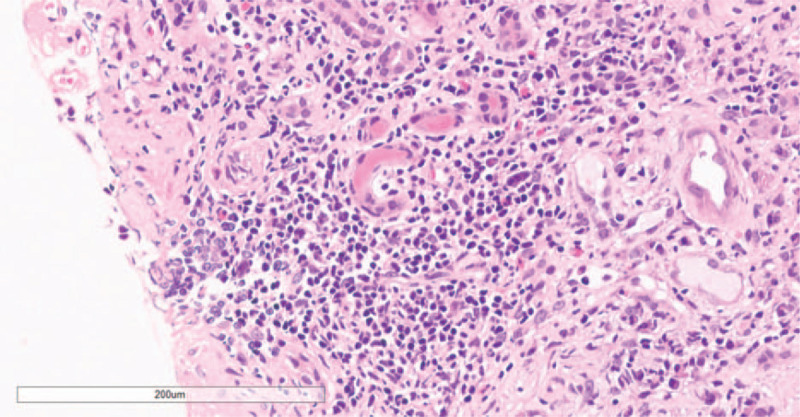
Hematoxylin and eosin staining of renal biopsy specimens showing chronic tubular-interstitial inflammation, diffuse atrophy of the renal tubular epithelium, extensive lymphocytic infiltration of the renal interstitial, and occasionally eosinophils (200×).

**Figure 3:**
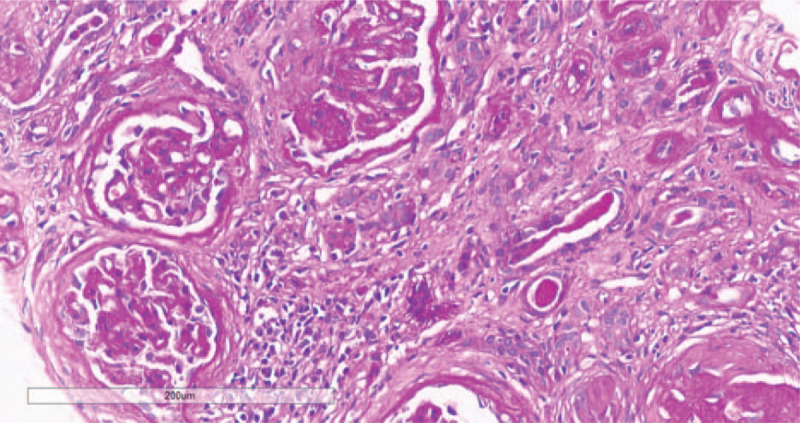
Periodic acid–Schiff staining showing glomerular ischemic changes; fibrosis can be seen around the balloon, and the capillary vasospasm is contracted by ischemia. Renal tubular epithelial cells showing vacuoles, granule degeneration, and a small amount of protein casts and diffuse atrophy. Renal interstitial showing lymphocytes and mononuclear cell infiltration with fibrosis, vitreous changed in the wall of the arterioles, increased thickness of the wall of small arteries, intimal hyperplasia, stenosis of the lumen, no cellulose-like necrosis (200×).

A renal biopsy on day 18 of hospitalization revealed severe and chronic injury to the renal tubules and interstitium, accompanied by glomerular ischemia and shrinkage. Immunohistochemical analysis showed that most renal interstitial lymphocytes were CD3^+^/MPO^+^ neutrophils with a small number of CD20^+^ lymphocytes and CD38^+^ interstitial cells. Furthermore, there was diffuse atrophy of kidney tubules, proliferation of interstitial collagen fibers, and infiltration of lymphocytes, and occasional presence of eosinophils (see Figs. 2 and 3 for details). Electron microscopy showed vacuolar degeneration of the capillary endothelium and narrowing of the lumen of capillaries.

Treatment with methylprednisolone infusion (40 mg qd) was initiated on day 7 of hospitalization, and the rash resolved after 1 week of therapy. The medication was switched from glucocorticoid to oral prednisone (40 mg qd) after 2 weeks of methylprednisolone therapy. Subsequently, a series of investigations demonstrated a decrease in the count and proportion of eosinophils to normal levels, an increase in urinary volume, a fall in serum creatinine to 262 to 276 μmol/L, and a glomerular filtration rate (GFR; estimated using the Modification of Diet in Renal Disease [MDRD] formula) of 25 mL/min. Dialysis was successfully discontinued after 3 weeks of steroid therapy (hemodialysis was given intermittently for a total of 10 sessions). The patient was discharged on day 28 (September 25, 2015) of hospitalization and prescribed prednisone tablets at an initial dose of 35 mg/day, with tapering of the dose by one tablet (5 mg) every 2 weeks. However, severe pulmonary infection and respiratory failure developed ∼2 months after the initiation of steroid therapy. The patient was admitted to hospital, the trachea was cannulated, and a diagnosis of invasive pulmonary aspergillosis (IPA) was made following bronchial brushing during bronchoscopy. Despite the initiation of anti-fungal therapy with caspofugin (Cancidas) and fluconazol, the patient exhibited progressive deterioration of renal function and died on December 8, 2015.

## 3 Discussion

While dermatologists usually focus on the varied cutaneous manifestations of DRESS, nephrologists are concerned with the renal injury that results from drug-induced acute interstitial nephritis (AIN). DRESS often affects the function of the renal tubules and interstitium and, thus, results in polyuria and glycosuria; however, the syndrome rarely results in kidney failure. Omeprazole-induced AIN was first described in 1992,^[14]^ and all PPIs currently on the market, including pantoprazole,^[15]^ omeprazole,^[16]^ rabeprazole,^[17]^ esomeprazole,^[18]^ and lansoprazole,^[19]^ have been reported to induce AIN. The incidence of PPI-induced AIN is 2 to 20 per 100,000^[7,20]^ and is not related to the drug dosage, suggesting the importance of individual factors, such as an allergic predisposition.^[20]^ The pathogenesis of PPI-induced DRESS is not fully characterized but may involve the deposition of a hapten with the drug (or its metabolite) in the tubulointerstitium, direct stimulation of the abnormal expression of T-cells, or Th1- and Th17-mediated inflammatory processes.^[21]^ Furthermore, genetic polymorphism of *CYP2C19* that slows the metabolism of PPIs is thought to increase the risk of acute renal injury.^[5,20,22]^

The case reported here relates to the recurrence of DRESS syndrome and impaired renal function after the repeated use of PPIs. On both occasions, the patient developed renal failure that required renal replacement therapy. Generally, renal injury occurs within 2 to 6 weeks after exposure to the causative drug. In this case, the patient relapsed 1 year after the initial episode of DRESS due to re-administration of a PPI, which, to the best of our knowledge, has not been described previously. The patient in this case report was found to have chronic interstitial nephritis, whereas the pathologic manifestations of PPI-induced acute renal injury are generally those of acute allergic intestinal nephritis.^[7,14–19]^

Acute renal injury caused by PPIs is histologically manifested as an infiltration of mixed inflammatory cells (lymphocytes, eosinophils, plasma cells, and isolated groups of neutrophils) in the renal interstitium.^[7,17,23]^ Geevasinga et al identified 18 cases of biopsy proven PPI-induced AIN causing AKI in a retrospective case review.^[17]^ A growing body of literature and case reports confirms that PPI therapy is linked to acute kidney injury, which can potentially lead to chronic kidney disease (CKD) and end-stage renal disease (ESRD).^[24–26]^

The pathologic changes underlying the development of chronic interstitial nephritis in the present case may have involved the initial occurrence of AIN and edema (during the first episode of PPI-induced DRESS), which then gradually progressed to renal interstitial fibrosis, tubular atrophy, and glomerular sclerosis.^[27]^ The pathological finding in the present case is in line with the role of acute injury in CKD, as reported in literature. The exact pathology of CKD caused by PPIs has not been previously reported for any case.

One of the factors that likely contribute to the development of chronic interstitial nephritis in patients with PPI-induced AIN is a missed or delayed diagnosis due to the atypical clinical manifestations of hypersensitivity reactions caused by PPIs. For example, fewer than half of the cases have fever, <10% develop a rash, about one-third manifest hypereosinophilia, and only 5% to 10% present with typical symptoms of a hypersensitivity reaction.^[28]^ Because it can take several weeks or even months to confirm a diagnosis of AIN after the development of symptoms, some patients inevitably develop chronic renal interstitial fibrosis before treatment can be initiated.^[28,29]^

It has been reported that renal function does not recover to the baseline in a substantial proportion of patients with PPI-induced AIN despite discontinuation of the causative drug and treatment with steroids.^[7]^ Furthermore, long-term administration of PPIs may increase the risk of chronic renal injury. For example, when compared with H2 receptor antagonists, long-term PPI use is associated with a higher risk of renal disease that progresses to CKD and ESRD.^[24,30,31]^ We suggest that renal biopsy may represent a useful technique for characterizing the pathological changes underlying the progression of AIN to chronic renal injury.^[30,32]^ Furthermore, the use of renal biopsy could help in increasing our understanding of the factors influencing the prognosis of renal disease caused by long-term PPI use and the ability of renal function to recover after discontinuation of the drug.

**Figure 4:**
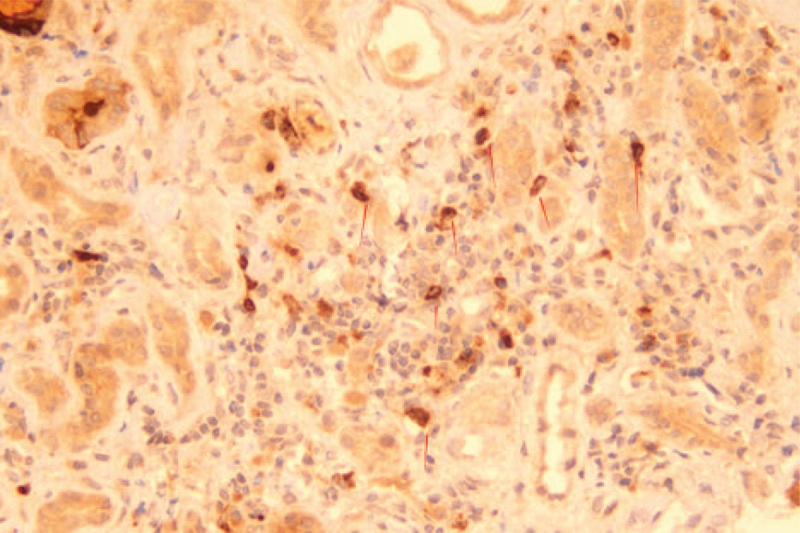
Myeloperoxidase staining of the renal interstitium. The arrow indicates the positively stained cells (200×).

There is also cross-reactivity in PPI-induced AIN, necessitating a skin prick test before the use of an alternative PPI and systemic desensitization for those who must use it.^[3]^ A recent case report described acute renal injury caused by the administration of two different PPIs (omeprazole first, pantoprazole later) to the same individual.^[33]^ The present case reported the prior use of three different PPIs (omeprazole, pantoprazole, and esomeprazole), suggesting that cross-reactivity may have contributed to the development of renal injury. Only two previous case reports have suggested that omeprazole^[34]^ and pantoprazole^[35]^ can induce ANCA-related vasculitis, and immunofluorescence microscopy of renal biopsy specimens demonstrated deposition of oligoimmune complex in a patient treated with pantoprazole.^[35]^ The renal biopsy specimens from the patient reported here were positive for IgM antibody but negative for other antibodies, such as IgG and IgA, which is consistent with the deposition of oligoimmune complex. However, the underlying mechanism is still unclear. In this case, the patient also tested positive for the anti-MPO antibody, which was deposited in the renal interstitium (see Fig. 4 for details). To the best of our knowledge, this is a novel finding that has not been described in previous studies.

The established treatment of drug-induced AIN involves discontinuation of the drug and administration of a glucocorticoid as an immunosuppressant.^[36,37]^ Early intervention with a glucocorticoid agent upon confirmation of renal pathology is thought to reduce the risk of renal interstitial fibrosis and facilitate the recovery of impaired renal function^[38]^ However, early diagnosis and intervention to prevent renal failure require close cooperation between physicians from different departments, in particular nephrology and pathology. Pathologic markers to guide therapy (such as the ratio of glomerular sclerosis to renal tubular atrophy, the degree of renal arteriolar occlusion, or the extent of interstitial fibrosis) are yet to be established. Nonetheless, it is envisaged that the development of standardized methods to evaluate the severity of interstitial nephritis and disease chronicity will facilitate decision-making by clinicians in the future.

PPIs are over-the-counter drugs that are widely used without prescription.^[28]^ Many clinicians have expressed concerns about the safety implications of the misuse of PPIs due to widespread availability of these drugs and a lack of regulation.^[20,39,40]^ PPIs are more frequently used by the elderly population than by the young people, which may contribute to a higher prevalence of AIN and CKD as well as a higher proportion of patients in need of dialysis.^[29]^

The following factors may significantly affect the clinical outcomes—lack of the awareness of PPI-induced AKI or other types of allergic manifestations, individual allergic idiosyncrasy, time of identification after the onset of PPI hypersensitivity reaction, therapeutic drug reaction to this syndrome.^[41]^

Most patients with PPI-induced DRESS can achieve a good prognosis if confirmation of the diagnosis, discontinuation of the PPI, and initiation of glucocorticoid-based therapy are performed in a timely manner.^[11]^ However, the early diagnosis and treatment of PPI-induced DRESS requires a multidisciplinary approach. The present case highlights the need for clinicians to remain on high alert for potential life-threatening complications when prescribing PPIs. Furthermore, renal biopsy may help in identifying the pathogenesis and facilitate timely intervention in patients who develop unexplained renal injury after starting a course of PPI therapy.

## Author contributions

**Conception and design:** Min Xia.

**Administrative support:** Min Xia, Xuelin He.

**Provision of study materials or patients:** Guanghui Ying, Xiapei Fei, Chenqin Zha, haogui Chen, Yishu Bao.

**Collection and assembly of data:** Qien He, Zhujun Wang,Min Xia.

**Data analysis and interpretation:** Qien He.

**Manuscript writing:** All authors.

**Final approval of manuscript:** All authors.
